# Myocardial strain imaging in Duchenne muscular dystrophy

**DOI:** 10.3389/fcvm.2022.1031205

**Published:** 2022-11-23

**Authors:** Conner C. Earl, Jonathan H. Soslow, Larry W. Markham, Craig J. Goergen

**Affiliations:** ^1^Weldon School of Biomedical Engineering, Purdue University, West Lafayette, IN, United States; ^2^Indiana University School of Medicine, Indianapolis, IN, United States; ^3^Division of Pediatric Cardiology, Department of Pediatrics, Vanderbilt University Medical Center, Nashville, TN, United States; ^4^Division of Pediatric Cardiology, Riley Children's Hospital, Indiana University Health, Indianapolis, IN, United States

**Keywords:** duchenne muscular dystrophy, strain imaging, cardiac magnetic resonance, pediatric cardiology, cardiac imaging, echocardiography, strain

## Abstract

Cardiomyopathy (CM) is the leading cause of death for individuals with Duchenne muscular dystrophy (DMD). While DMD CM progresses rapidly and fatally for some in teenage years, others can live relatively symptom-free into their thirties or forties. Because CM progression is variable, there is a critical need for biomarkers to detect early onset and rapid progression. Despite recent advances in imaging and analysis, there are still no reliable methods to detect the onset or progression rate of DMD CM. Cardiac strain imaging is a promising technique that has proven valuable in DMD CM assessment, though much more work has been done in adult CM patients. In this review, we address the role of strain imaging in DMD, the mechanical and functional parameters used for clinical assessment, and discuss the gaps where emerging imaging techniques could help better characterize CM progression in DMD. Prominent among these emerging techniques are strain assessment from 3D imaging and development of deep learning algorithms for automated strain assessment. Improved techniques in tracking the progression of CM may help to bridge a crucial gap in optimizing clinical treatment for this devastating disease and pave the way for future research and innovation through the definition of robust imaging biomarkers and clinical trial endpoints.

## 1. Introduction

Duchenne muscular dystrophy (DMD) is a severe, progressive, X-linked genetic disease that affects roughly 1 in every 5,000 live male births (1–4). DMD results from a mutation in the gene which encodes for the protein dystrophin, leading to a progressive impairment of muscle function (5, 6). A lack of dystrophin leads to a loss of sarcolemma integrity triggering muscle degradation and inflammation followed by necrosis, fibrosis, and fibro-fatty replacement (7, 8). While the average lifespan is now entering the third decade, significant morbidity exists with loss of ambulation by age 13 and death for some in late teens or twenties from respiratory or cardiovascular complications (9, 10). While all DMD patients have inexorably progressive CM, the age of onset and clinical progression of cardiac complications are varied. However, early diagnosis and treatment of CM in DMD patients have been shown to improve both quality and length of life (1, 9).

Cardiac imaging provides both a method for early identification of DMD CM and an opportunity for personalized treatment planning. While advances in imaging technology and data processing have allowed for significant improvements in the diagnosis of DMD CM, there is still more work to be done to fully characterize this disease process using imaging-based metrics. Transthoracic echocardiography (TTE) and cardiac magnetic resonance (CMR) are primarily used in the assessment of cardiac function in DMD patients, and each has distinct advantages in this population. TTE is faster, cost-effective, more accessible, and generally easier to perform on a younger patient population. However, as the disease progresses, TTE is often limited by poor echocardiographic windows due to scoliosis and fat deposition (11–13). CMR is not limited by acoustic windows and it allows for accurate volumetric assessment and tissue characterization through methods such as T1 mapping and late gadolinium enhancement (LGE) (13, 14). It is for these reasons that CMR has become the gold standard for evaluating CM in the DMD patient population for its ability to provide superior diagnostic capability (15–19). Additional cardiac imaging modalities such as magnetic resonance spectroscopy, single-photon emission computed tomography, and x-ray computed tomography can be used for assessment, but are not commonly used in assessment of CM in DMD (20–23). In the DMD patient population, heart function abnormalities such as changes in left ventricular ejection fraction (LVEF) are often masked in the early stages of CM as these patients may be wheelchair-bound and incapable of robust physical activity from skeletal muscle degradation. Thus, metrics such as cardiac image-based strain mapping may be especially useful in this population as they could be more sensitive to subtle mechanistic changes in cardiac disease progression.

## 2. Myocardial strain

The mechanics of cardiac contraction can provide a wealth of information regarding cardiac fitness. The heart wall consists of a group of continuous myocardial fibers oriented in various patterns to produce contraction. For example, many fibers in the subendocardium follow a right handed helical orientation, while in the subepicardium the fibers follow a left handed helical formation with midmyocardial fibers aligning in the circumferential direction (24). The orientation of these fibers is important in determining the mechanical properties of the heart wall as well as the magnitude and principal direction of strain. Subtle changes in these properties can be indicative of cardiac pathology or disease exacerbation (25, 26). This is particularly important in the progression of DMD CM where initial fibrosis is often first seen in the basal subepicardium (21) affecting circumferentially oriented fibers making changes in basal circumferential strain (E_θθ_) an early predictor of disease (15, 17).

Put simply, cardiac strain is a measurement of the “stretch” or deformation of the heart wall and is often measured relative to the heart at end-diastole when the ventricle is filled with blood. This simplification is useful, but it is not a perfect definition as pre-strain and pre-stress are present in the myocardium at end-diastole due to the load imposed by the blood and the structural properties of cardiac tissue. Strain is a tensor quantity with normal and shear components, however, a simpler linear approximation of strain, referred to as engineering strain, can be used. The linear approximation of strain is calculated as a change in relative length compared to a reference length and typically assumes strain levels are small (typically below 5%) and may not be as accurate at higher values (27).

From a mechanics perspective, a more robust definition of strain can be calculated in the Lagrangian reference frame to account for multi-directional components of deformation. The 3-dimensional Green-Lagrange strain tensor (**E**) depends on the 3D deformation gradient tensor (**F**) and the identity matrix (**I**) (28, 29) shown below in Equation (1).


(1) $$\begin{array}{l} {\text{E}}_{ij}=\left[\begin{array}{ccc} {\text{E}}_{11} & {\text{E}}_{12} & {\text{E}}_{13}\\ {\text{E}}_{21} & {\text{E}}_{22} & {\text{E}}_{23}\\ {\text{E}}_{31} & {\text{E}}_{32} & {\text{E}}_{33} \end{array}\right]=\frac{1}{2}\left({\text{F}}_{ij}^{T}\cdot {\text{F}}_{ij}-{\text{I}}_{ij}\right)\text{; for i, j}=1,2,3 \end{array}$$

This generic formula can be adapted to describe deformation of the myocardium with respect to cylindrical coordinates. Given the complex three-dimensional shape of the heart, this is particularly helpful with cardiac strains often described using three directional components (radial, E_*rr*_; longitudinal, E_*ll*_; and circumferential, E_θθ_), with three corresponding shear components of strain used to define twist or rotational motion (E_*rl*_, E_*rθ*_, E_*lθ*_) (30, 31). Additional strain metrics unique to three-dimensional imaging can also be defined, including surface area strain (E_*A*_). Surface area strain is defined by the relative change in the size of the endocardial or epicardial surface area of the ventricle from a three-dimensional segmentation (32).

## 3. Transthoracic echocardiography

### 3.1. One- and two-dimensional strain imaging

**Figure 1:**
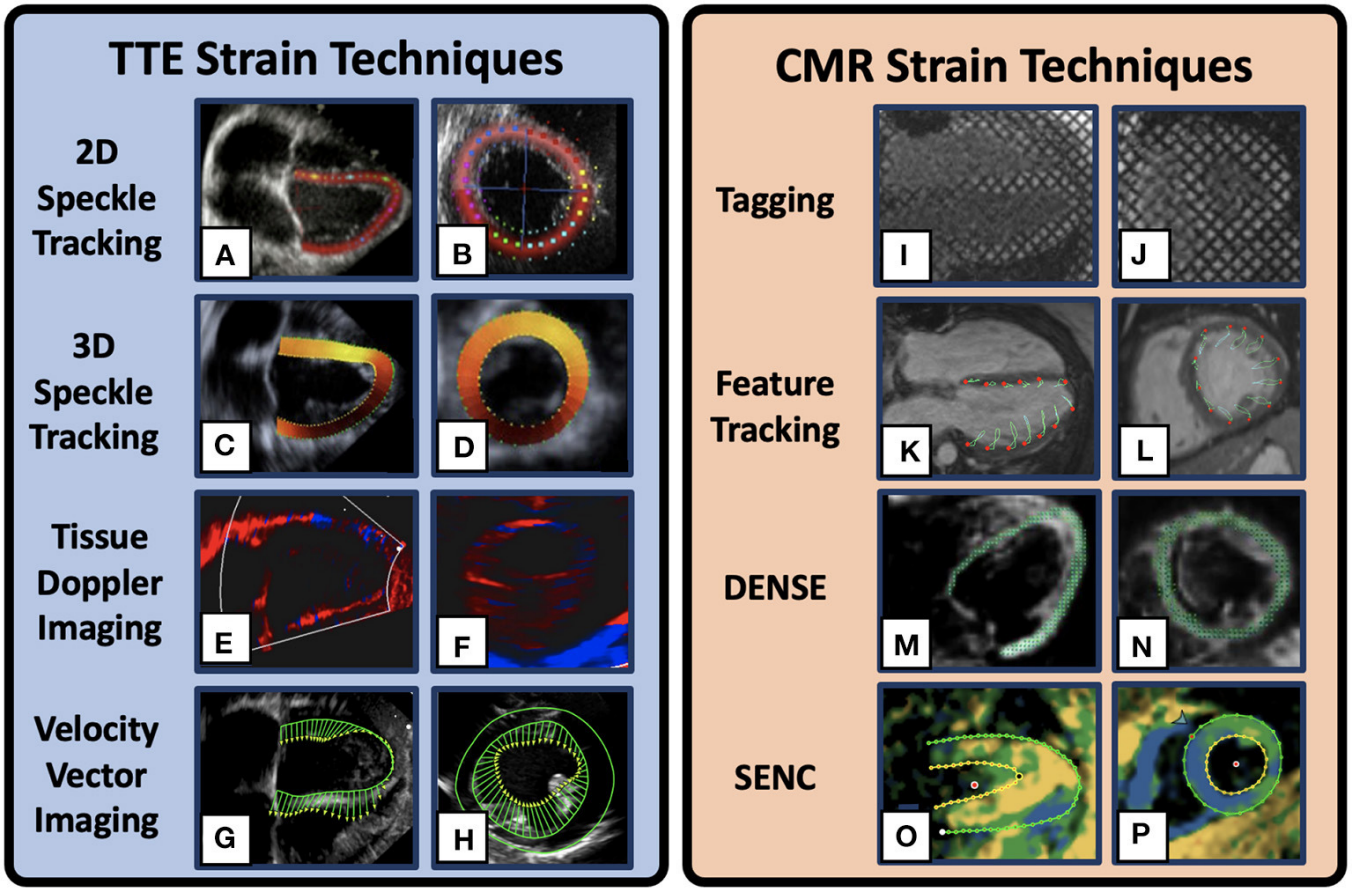
Example Cardiac Strain Imaging Techniques. 2D speckle tracking Transthoracic echocardiogram (TTE) image in the 4-chamber **(A)** and mid-ventricular short-axis **(B)**[adapted from Mele et al. (33)]. 3D speckle tracking TTE images in the 4-chamber **(C)** and short-axis **(D)**[adapted from Maffessanti et al. (34)]. Tissue Doppler imaging TTE images in the 2-chamber long-axis **(E)** and short axis **(F)** in a DMD patient. Velocity vector TTE images in the 4-chamber **(G)** and short axis **(H)** in a DMD patient. Cardiovascular magnetic resonance (CMR) strain method depicting myocardial tagging in the 4-chamber **(I)** and mid-ventricular short-axis **(J)** views in a DMD patient. CMR feature tracking example images in the 4-chamber **(K)** and short axis **(L)** views. Displacement Encoding with Stimulated Echoes (DENSE) example images in the 4-chamber **(M)** and short-axis **(N)** views [adapted from Cao et al. (35)]. Strain Encoded example images in the 4-chamber **(O)** and short-axis **(P)** view plane in a DMD patient. Multiple visualizations are available for each technique and the images presented here are only representative.

Strain is measured from clinical TTE using three primary techniques: 1) speckle tracking echocardiography (STE) (Figures 1A,B, Table 1), 2) velocity vector imaging (VVI) (Figures 1G,H, Table 1), and 3) tissue Doppler imaging (TDI) (Figures 1E,F, Table 1). Beginning at end-diastole, speckle tracking echocardiography relies on a computational algorithm to track the black and white speckle pattern of the myocardium as it deforms throughout the cardiac cycle. By tracking the motion of these speckles through space and time around a given point in the tissue, one can derive the “motion description” of strain, i.e., Lagrangian strain, in which deformation of the myocardium is tracked from a fixed reference point at end-diastole (30). Strain mapping with 2D-STE has been shown to be useful for early detection of cardiac dysfunction retrospectively in DMD patients and prospectively in canine models of DMD even prior to gross changes in LVEF (12, 37, 40, 41). In one notable recent study of 38 ambulatory boys with DMD (mean age 8.8 ± 1.6 years) 9% showed reduced global strain (< 18%) on 2D-STE prior to clinical features suggestive of cardiac dysfunction (41).

**Table 1:** Stain imaging technique comparison in DMD.

**Modality**	**Basic description**	**Advantages**	**Disadvantages**	**Application In DMD**
**Cardiac Magnetic Resonance Strain Techniques**				
Tagging/HARP	Tracks grid of radio-frequency labeled tags on myocardium	Commonly used, good reproducibility, extensive validation	Low spatial resolution, post-processing, fading tags, no standardization, additional scan time	(15, 17)
CMR-FT	Tracks brightness, tissue contrast throughout cycle	No additional scanning needed, regional and global strain measurements	Regional strain less reproducible, motion artifacts, sedation may be required in young children	(36)
SENC, DENSE, TPM, HARP	Strain measurement directly from specific MR sequences	High spatial and temporal resolution, good accuracy	Additional acquisitions, post-processing, not well-studied in DMD	(15, 17)
**Echocardiography Strain Techniques**				
2D-STE	Tracks image texture (speckles) in the myocardium	Angle independent, semi/fully automatic analysis, less noise than TDI	Through plane motion, poor imaging windows in DMD, inter-vendor differences	(37)
3D-STE	Tracks image texture (speckles) in 3D image of the myocardium	Measures deformation in 3D, no geometric assumptions, LV rotational deformation possible, E_*A*_ estimate possible	Lower spatial and temporal resolution compared to 2D-STE, inter-vendor differences, no standardization	(38)
TDI	Measures tissue velocity gradients which are then integrated to derive strain	High temporal resolution, fast assessment of single region	Angle dependent, 1D, comprehensive LV assessment cumbersome, noisier than STE	(39)
VVI	Integrates 2D tissue velocity gradients to derive strain	2D, reproducible, angle independent	Image quality dependent, vendor variability, no standardization	(12)

STE, speckle tracking echocardiography; DTI, Doppler Tissue Imaging; VVI, Velocity Vector Imaging; CMR-FT, Cardiac Magnetic Resonance Feature Tracking; SENC, Strain ENCoded; DENSE, Displacement Encoding with Stimulated Echoes; TPM, Tissue Phase Mapping; HARP, HARmonic Phase imaging.

Another method for estimating strain is tracking the relative velocity of each speckle throughout the cardiac cycle with a fixed spatial reference frame. This “spatial description” of strain, i.e., Eulerian or “normal” strain (30). Both TDI and VVI rely on velocity tracking within the tissue and calculate the Eulerian strain. However, in practice, many imaging software packages have the capability of converting between Eulerian and Lagrangian frames of reference (42). TDI specifically utilizes a one-dimensional approach to estimate relative changes in velocity using Doppler ultrasound, while VVI utilizes a two-dimensional B-mode image to track speckle velocities. Both modalities have a relatively high temporal resolution, though TDI is angle-dependent and is only capable of analyzing velocities in one region of the myocardium at a time making comprehensive LV assessment cumbersome. Despite these limitations, both TDI (39) and VVI (12) have been used successfully to show strain differences in small DMD populations compared to age-matched controls (*n* = 32 and *n* = 24 respectively).

### 3.2. Three-dimensional strain imaging

Advances in ultrasound technology in recent decades have allowed for three-dimensional analysis of myocardial mechanics. 3D imaging has shown good agreement with other techniques and reduced interuser variability, though its use clinically is still limited and more research is needed to characterize its incremental advantages over conventional 2D measurements (43). While 2D-STE is prone to error propagation and relies on geometric assumptions regarding the left ventricle, 3D-STE is more reproducible in both clinical and experimental models (44, 45) (Figures 1C,D). In addition, 3D imaging allows for the calculation of unique strain metrics such as surface area strain (E_*A*_). In DMD patients, strain mapping with 3D-STE can detect early clinical progression despite these patients having normal LVEF (38). Yu et al. demonstrated that (E_*A*_) derived from 3D-STE had an 85.7% sensitivity and a 71.0% specificity for differentiating DMD patients (*n* = 56) from controls (*n* = 31; (38); Table 1). While these metrics show improvement over 2D-STE-derived metrics, this method still suffers from limited echocardiographic windows, low spatial and temporal resolution, relatively small study populations, and inter-vendor differences (11, 32, 46–48).

## 4. Cardiac magnetic resonance strain imaging

There are a variety of methods for estimating strain using CMR. Given the breadth of this field, this review focuses on techniques shown to have utility in DMD CM assessment including CMR-tagging (Figures 1I,J), HARmonic Phase (HARP) analysis, and CMR-Feature Tracking (CMR-FT) (Figures 1K,L), in addition to a brief discussion of additional sequences such as Tissue Phase Mapping (TPM), Displacement Encoding with Stimulated Echoes (DENSE) (Figures 1M,N), and Strain Encoded MRI (SENC) (Figures 1O,P) that are useful strain techniques, but that are currently less studied in the DMD population. The following reviews on strain imaging using CMR are excellent and provide more comprehensive descriptions of these techniques outside of DMD-specific applications (31, 49–53).

### 4.1. Tagging

CMR tagging is a strain estimation method that utilizes parallel radiofrequency pulses to impose orthogonal planes of saturated myocardial magnetization to rapidly create a grid of dark saturated pixels overlaying the myocardium at end-diastole [Figures 1I,J; (50, 54)]. These tags, or lines of saturation, remain throughout the cardiac cycle and fade according to the strength of the magnetic field. The deformation of this grid pattern can be tracked and analyzed using post-processing software to estimate regional and global strain. This method allows for longitudinal and circumferential strain estimates, but is often not reliable for radial strain estimates given the large spacing of the grid pattern leading to low resolution to estimate radial deformation (55).

HARmonic Phase (HARP) analysis is a commonly used strain imaging technique used in a large number of CMR post-processing software packages (55–57). HARP analysis in conjunction with CMR tagging has been used to estimate strain in DMD patients establishing strain as a reliable method to stratify cardiac disease severity (15, 17). The HARP technique relies on isolating one Fourier component of the amplitude modulated data and tracks the phase on a pixel-by-pixel basis (56). This technique allows for more rapid and reproducible strain estimation. Some drawbacks to the HARP method include a relatively low spatial resolution, tag fading, additional scan time, and a lack of standardization (50, 54).

### 4.2. Feature tracking

CMR-feature tracking (CMR-FT) is another deformation estimation technique that utilizes characteristic features in a CMR scan to estimate strain (Table 1). A spatial deformation map is produced by tracking tissue edges, brightness, and homogeneity throughout the cardiac cycle. However, given the relative homogeneity within the myocardium in conventional CMR images, regional estimates of strain are often misrepresented and there is additional work needed to improve these estimations. Despite these drawbacks, CMR-FT does not require any additional scan time as it does not need any specialized sequences, unlike most other CMR strain techniques. Additionally, in DMD patients, CMR-FT has been shown to detect morphologic changes in the absence of late gadolinium enhancement (LGE) as well as detect changes between DMD and controls not seen using 3D-STE (36). Another study by Raucci et al. showed that CMR-FT in DMD could be used to detect the presence or absence of LGE (OR 2.6[1.1,6.0], *p* = 0.029, and OR 2.3[1.0,5.1], *p* = 0.049, respectively), though the authors suggest these metrics should not replace LGE when contrast can be administered safely (58).

### 4.3. Other

DENSE, SENC, and TPM are additional methods for strain estimation using CMR but are less studied in DMD populations (Figures 1M–P, Table 1). DENSE, SENC, and TPM techniques all rely on specialized image sequences that take additional scan time, but they produce relatively high spatial and temporal resolution needed for strain estimates. DENSE is generally accepted as one of the most accurate and reproducible methods for strain imaging. DENSE uses a stimulated echo to produce the phase information that is directly proportional to tissue displacement. Through the analysis of directional-encoded phase images, the Lagrangian displacement fields can be produced to estimate strain. A recent study showed that regional circumferential strain as measured by DENSE CMR in healthy pediatric controls and DMD patients was both accurate and reproducible (ICC>75%) (59).

SENC, like HARP, uses radiofrequency pulses to create parallel tagged lines with out-of-plane phase encoding gradients to estimate strain. Tracked frequency changes can then be visualized to show contracting and non-contracting tissue by generating a high-resolution color-coded strain map (Figures 1O,P). While SENC has been used to visualize strain changes in DMD in research studies (14), to our knowledge, comprehensive studies showing its clinical use are still forthcoming. Finally, TPM is a technique that derives spatial deformation and strain directly from the pixel phase to encode velocity from each image. TPM has been shown to be useful for comprehensive analysis of myocardial motion with a high spatial and temporal resolution in pulmonary hypertension (60), obesity (61), measuring myocardial torsion (62), and identifying sex and age-specific differences in adults (63). TPM, SENC, and DENSE are not currently being widely used clinically or in DMD specifically, but they are being studied extensively in research settings (57, 64, 65) and in the future may provide additional value for assessing the rate of CM progression in DMD.

## 5. Machine learning and the future of cardiac imaging in DMD

The field of machine and deep learning has been progressing rapidly and is the fastest-growing approach for cardiac image segmentation in recent years (66). Fully convolutional neural networks, and in particular the U-Net network structure, have been particularly powerful in automating biomedical image segmentation and analysis (67, 68). These techniques, however, are susceptible to bias and rely on a large training dataset for “ground-truth” analysis. Although deep learning techniques have been applied to DMD phenotyping through genetic classification (69), to our knowledge, no one has applied these techniques to DMD cardiac image segmentation and patient classification and prognosis. This constitutes a promising area for future study and innovation.

### 5.1. Techniques

The type of machine and/or deep learning technique being used depends on the task that is being investigated (e.g., image classification, image segmentation, image enhancement). In the context of strain analysis, one common approach is to segment the cine image sequence followed by post-processing deformation analysis. The most common architecture used in medical image segmentation is a convolutional neural net (CNN) (70). A typical CNN functions by applying convolution filters to images followed by pooling layers which downsample the image data and allow for the CNN to incorporate contextual information. While this downsampling lowers image resolution, upsampling with concatenation allows for image resolution to be restored, thus allowing for the neural net to incorporate both low- and high-resolution features. The prime example of this type of CNN is a U-Net, so-called for its architecture incorporating convolution, pooling, and upscaling used for feature identification. The U-Net architecture functions by inputting a batch set of CMR images followed by feature classification. Through each iteration, multiscale features are learned through a series of convolutional layers and max-pooling operations. These learned features are then combined through upsampling and convolution to generate pixel-wise predictions for the selected class features (i.e., left ventricle, wall, right ventricle, etc. (67). The U-Net architecture has been shown to perform remarkably well in segmenting 2D and 3D CMR datasets (66, 67). These segmentations can in turn be used for rapid and automated cardiac strain analysis.

### 5.2. Feasibility and limitations in DMD

Numerous studies have shown the feasibility and efficacy of a deep learning algorithm using CMR data for automatic segmentation and detection of LV geometry and function (66, 67, 71, 72). Though this type of analysis has not been applied to a registry of DMD patient imaging data, future applications of these methods and algorithms could be used to greatly benefit the early identification of cardiac dysfunction and progression rate to aid in treatment planning. In addition, the use of these techniques on a large cohort of DMD patients could allow for generalizability of findings to other populations of interest such as female carriers of DMD who only exhibit subtle cardiac dysfunction (73).

A limitation of the deep learning approach is that the chosen algorithm may be susceptible to perturbations in image quality and artifacts, especially at the base and apex of the heart. One such example is a breathing artifact in CMR scans that appears as sections of myocardium offset from the rest of the scan, a problem especially pervasive in the younger DMD population (74). An alternative approach to overcome variability in image acquisition and artifacts could be to incorporate different available views from CMR imaging datasets (four-chamber long axis, two-chamber long axis, short axis) to correct for these abnormalities. Another potential limitation is the generalizability of this deep learning network to CMR images taken at a variety of locations where different imaging approaches, magnets, gradients, or magnetic field strengths may affect the quality of segmentation. Alternative structures and data processing such as the application of recurrent neural networks, data normalization, or data augmentation might improve the generalizability of these algorithms as demonstrated by Chen et al. (66, 67).

## 6. Conclusion

This literature assessment contextualizes cardiac strain imaging techniques in the setting of DMD CM. Here we reviewed the general pathophysiology of DMD-associated CM, discussed an overview of the most relevant imaging techniques, and reviewed the cardiac strain imaging methods generally and those that are important for characterizing DMD. We concluded by examining potential future applications that may fill these gaps with a particular focus on machine learning and emerging methods in artificial intelligence that could be used to guide treatment.

## Author contributions

CE, CG, JS, and LM helped with the conception, literature review presented in this article, helped draft, and critically revise the manuscript providing intellectual content. JS provided protected access to example CMR and ultrasound images. LM and JS provided key DMD-specific clinical expertise and expert imaging guidance. All authors approved the final version of this manuscript.

## Funding

This publication was supported by the Ackerman/Nicholoff Family (Indianapolis, IN) (LM), Fighting Duchenne Foundation and the Fight DMD/Jonah and Emory Discovery Grant (Nashville, TN) (JS), the Food and Drug Administration Orphan Products Grant R01FD006649 (JS), the National Center for Research Resources, Grant UL1 RR024975-01, and is now at the National Center for Advancing Translational Sciences, Grant 2 UL1 TR000445-06 (Bethesda, MD) (JS), and the National Heart, Lung, and Blood Institute of the National Institutes of Health under award Number K23HL123938 and R56HL141248 (Bethesda, MD) (JS) and F30HL162452 (CE). This publication was also made possible with support from Grant Number, UL1TR002529 (S. Moe and S. Wiehe, co-PIs) from the National Institutes of Health, National Center for Advancing Translational Sciences, Clinical and Translational Sciences Award (CE) and the Leslie A. Geddes Endowment at Purdue University (CG). Publication of this article was funded in part by Purdue University Libraries Open Access Publishing Fund. The content is solely the responsibility of the authors and does not necessarily represent the official views of the National Institutes of Health.

## Conflict of interest

The authors declare that the research was conducted in the absence of any commercial or financial relationships that could be construed as a potential conflict of interest.

## Publisher's note

All claims expressed in this article are solely those of the authors and do not necessarily represent those of their affiliated organizations, or those of the publisher, the editors and the reviewers. Any product that may be evaluated in this article, or claim that may be made by its manufacturer, is not guaranteed or endorsed by the publisher.
